# A Compact High-Isolation Four-Element MIMO Antenna with Asymptote-Shaped Structure

**DOI:** 10.3390/s23052484

**Published:** 2023-02-23

**Authors:** Aiting Wu, Yingxiang Tao, Pengquan Zhang, Zhonghai Zhang, Zhihua Fang

**Affiliations:** School of Electronics and Information, Hangzhou Dianzi University, Hangzhou 310018, China

**Keywords:** UWB antenna, multiple-input multiple-output, high isolation, asymptote-shaped, polarization diversity

## Abstract

The demand for high-speed wireless communication systems has led to the development of ultrawide-band (UWB) antennas with a compact size and high performance. In this paper, we propose a novel four-port multiple-input multiple-output (MIMO) antenna with an asymptote-shaped structure that overcomes the limitations of existing designs for UWB applications. The antenna elements are placed orthogonally to each other for polarization diversity, and each element features a stepped rectangular patch with a tapered microstrip feedline. The unique structure of the antenna significantly reduces its dimensions to 42 × 42 mm^2^ (0.43λ×0.43λ@ 3.09GHz), making it highly desirable for use in small wireless devices. To further enhance the antenna’s performance, we use two parasitic tapes on the ground plane at the back as decoupling structures between adjacent elements. The tapes are designed in a windmill shape and a rotating extended cross shape, respectively, to further improve the isolation. We fabricated and measured the proposed antenna design on a single-layer substrate (FR4) with a dielectric constant of 4.4 and a thickness of 1 mm. The measured results show that the impedance bandwidth of the antenna is 3.09–12 GHz, with an isolation of −16.4 dB, an envelope correlation coefficient (ECC) of 0.02, a diversity gain (DG) of 9.991 dB, an average total effective reflection coefficient (TARC) of −20 dB, an overall group delay value less than 1.4 ns, and a peak gain of 5.1 dBi. Although there may be some antennas that have better performance in one or two specific aspects, our proposed antenna has an excellent trade-off among all the antenna characteristics including bandwidth, size, and isolation. The proposed antenna also exhibits good quasi-omnidirectional radiation properties, making it well-suited for a range of emerging UWB-MIMO communication systems, particularly in small wireless devices. In summary, the compact size and ultrawide-band capabilities of the proposed MIMO antenna design, coupled with its improved performance compared to other recent UWB-MIMO designs, make it a promising candidate for 5G and next-generation wireless communication systems.

## 1. Introduction

The tremendous growth and rapid development in the field of wireless communication have prompted people to seek new technologies that can provide large and fast data transmission. UWB technology has attracted a great deal of attention due to its wide bandwidth, high data transmission rate, and strong anti-interference capability. Since the Federal Communications Commission (FCC) released 3.1–10.6 GHz for unauthorized access and commercial use [1], UWB has evolved into a unique technology within wireless communications, radar, smart home wearable devices, and medical applications [2,3,4]. The relatively low radiation power of UWB systems limits their transmission distance. However, the combination of UWB and MIMO technologies has been shown to be a feasible solution for improving the anti-multipath fading capability [5,6]. By utilizing multiple antennas on both transmitters and receivers, MIMO technology enhances the channel capacity of wireless communication systems, thereby significantly extending the transmission distance of UWB systems. 

For the study of UWB-MIMO antennas, wideband and high isolation are important research directions. The challenge of designing UWB-MIMO antennas is the coupling between antenna units over small sizes, especially in compact devices with limited space. The distance between units of a UWB-MIMO antenna should be half the wavelength of the minimum at the lowest operating frequency of the UWB band to meet the isolation requirements, which results in a large overall dimension of the antenna. Therefore, appropriate decoupling structures between antenna elements are proposed to improve the isolation. Several designs have been proposed for decoupling MIMO antennas. In [7,8,9], good isolation is obtained by orthogonal polarization and optimizing spacing between elements without any decoupling structure, increasing the isolation among the MIMO antenna elements. In [10], mutual couplings among the antenna elements are significantly reduced by using a two-sided symmetric layout and introducing the symmetric orthogonal and separated four-directional staircase-shaped structure, achieving isolation of less than −22 dB. In [11], high isolation and polarization diversity are achieved by placing the four microstrip-fed lines perpendicular to each other, and a parasitic strip is employed as a decoupling structure between adjacent microstrip-fed lines to further improve isolation, with the isolation of the antenna being higher than −15 dB in the whole UWB frequency band. In [12], a periodic linear network formed by four units of a square ring resonator (SRR) is inserted between the two antenna elements designed with an isolation of higher than −25 dB. A ground stub and a single-column Electromagnetic Bandgap (EBG) structure in between the two radiating patches result in very low mutual coupling in the designed antenna with a low mutual coupling of less than −25 dB [13]. In [14], a wideband neutralization line is placed between two MIMO elements, and the designed UWB MIMO antenna covers the band of 3.1–5 GHz with an isolation of higher than −22 dB. In [15], a special-material MIMO antenna is presented to increase isolation. In [16], the antenna design employs a hybrid isolation enhancing (inverted-L stubs) and miniaturization technique (CSRR), with mutual coupling lower than −15 dB. As a flexible method, defective grounding structures (DGS) are well-used in MIMO antenna decoupling [17,18,19,20]. In [21,22], two antennas address the coupling using stubs that not only reduce mutual coupling by acting as a reflector to separate the radiation of the elements but also act as a radiator to produce resonances. Additionally, the low coupling can be achieved by designing the ground of the MIMO antenna [23,24]. However, there is always a trade-off among antenna size, isolation, complexity, and cost in all of these proposed methods and techniques. In [8,25], the antennas improve isolation by using polarization diversity. The dimensions are 50 × 39.8 mm^2^ and 60 × 60 mm^2^, respectively, which are too large to integrate. In [11], the antenna meets the UWB band requirements with a small size, but the isolation is just less than −15 dB. The two antennas described in [26,27] achieve high isolation, but the fabricating process of the design is relatively complex, and antenna tuning is difficult. In [28,29,30], Rogers and Taconic high-frequency materials are used to achieve high isolation and small size, but these two materials are expensive. The proposed design achieves a favorable trade-off compared to existing designs.

In this study, a compact four-port UWB-MIMO antenna with high isolation and low cost is proposed for ultra-wideband wireless communication networks. The four elements of the proposed antenna are identical and placed perpendicular to each other on the substrate to greatly reduce the overall size. On the front side, each element consists of a stepped rectangular radiation patch and an asymptote-shaped microstrip feeder to expand the bandwidth. On the back side, a windmill shape and rotating extended cross shape are used to reduce the mutual coupling among the radiating elements. Simulated and measured results indicate that the proposed UWB-MIMO antenna has a wide impedance bandwidth and high isolation. Additionally, the antenna’s radiation characteristics, MIMO diversity characteristics, and peak gain characteristics are investigated and analyzed. The highlight of the proposed antenna is the introduction of the asymptote-shaped structure, which is incorporated into the overall structure, including the rectangular patch, microstrip feeder, and windmill-shaped decoupling structure. This expansion of the bandwidth of the antenna improves isolation while effectively reducing the antenna’s size. The main contributions of the proposed design include:

- Adoption of a stepped rectangular radiation patch and placement of the four elements orthogonally to each other to achieve a compact antenna size and improve antenna isolation.
- Use of an asymptote-shaped microstrip feeder to expand the bandwidth.
- Use of windmill and rotating extended cross-shaped decoupling structures to improve antenna isolation.
- Combination of all the above optimizations to achieve good characteristics in terms of bandwidth, size, and isolation.

The rest of this paper is organized as follows: Section 2 presents the antenna design and introduces methods to improve the bandwidth, size, and isolation. Section 3 analyzes and discusses the antenna’s performances, including return loss, isolation, peak gain, diversity gain (DG), ECC, TARC, group delay, and far-field radiation pattern characteristics. The conclusion is drawn in Section 4.

## 2. Antenna Design

**Figure 1:**
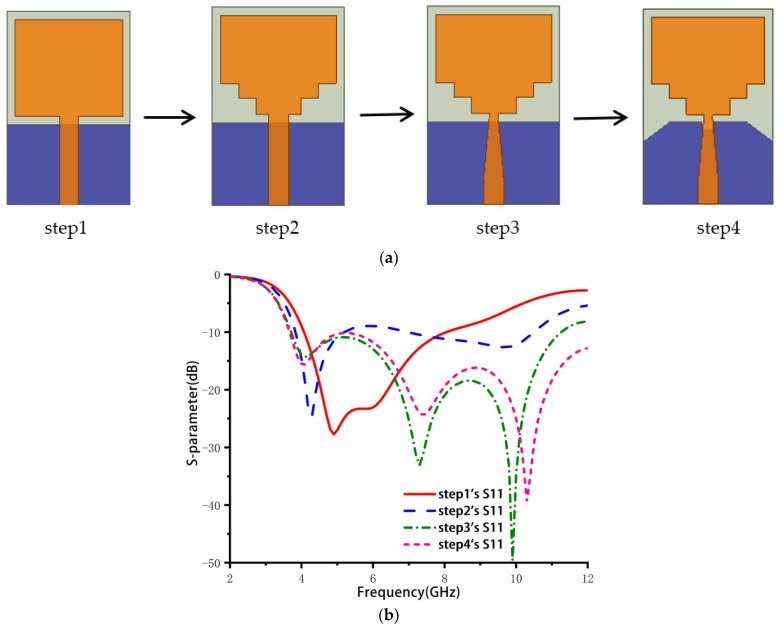
The geometry of steps 1 to 4 and simulated S parameters: (**a**) Geometry, (**b**) S parameters.

Figure 1 depicts the four steps of the antenna element design and their corresponding reflection coefficients. Step 1 represents the original antenna, which consists of a rectangular patch, a ground plate, and a 50 Ω microstrip feeder. Its impedance band ranges from 4.07 to 8.05 GHz. To increase the bandwidth, small rectangles are cut at the bottom of the rectangular patch to make it gradually step in step 2. This increases the current path length and generates new resonance points, resulting in a dual-band performance of 3.83–5.19 GHz and 6.97–10.5 GHz. However, this bandwidth is not yet sufficient to cover the entire UWB frequency band. In step 3, the feeder is cut and becomes a stepped rectangle, achieving a return loss of less than −10 dB in the 3.71–11.26 GHz range. Finally, the ground plate is cut in step 4 to further improve the bandwidth to 3.6–12 GHz.

The primary design of the antenna consists of a substrate, a rectangular patch, a ground plane, and a 50 Ω microstrip feedline. To calculate the preliminary length (L) and width (W) of the patch for a rectangular microstrip antenna at a given operating frequency, Equations (1)–(3) below can be used.

(1) $$W=\frac{c}{2f}{\left(\frac{\varepsilon_{r}+1}{2}\right)}^{-\frac{1}{2}}$$

*ε*_r_, *c*, and *f* in Equation (1) represent the substrate’s relative permittivity, the speed of light, and the operating frequency. To achieve excellent impedance matching, the optimal choice of W can be determined. The length of the radiation patch can be calculated using two methods: One is a rough calculation that takes the length as 0.5$\lambda_{e}$, and $\lambda_{e}$ can be calculated using Equation (2); the other method considers the edge-shortening effect, and the actual radiating element length L can be calculated using Equation (3).

(2) $$\lambda_{e}=\frac{c}{f\sqrt{\varepsilon_{e}}}$$

(3) $$L=\lambda_{e}-2\Delta L$$

where $\lambda_{e}$, $\varepsilon_{e}$, and ΔL stand for the effective dielectric constant, the guided-wave wavelength within the medium, and the change in the patch’s size due to its fringing effect. The effective relative permittivity and the change in the size of the patch can be calculated using Equations (4) and (5), where ‘*h*’ is the substrate’s height. The patch’s initial parameters are W = 14.6 mm and L = 17.4 mm, based on $\varepsilon_{r}$= 4.4 and h = 1 mm in Equations (1)–(5). This calculated value is used as a reference value. As shown in Figure 1a, the antenna operates at 4.5 GHz using the initial patch. Subsequently, a wide bandwidth was achieved by modifying the rectangular patch and ground joint.

(4) $$\varepsilon_{e}=\frac{\varepsilon_{r}+1}{2}+\frac{\varepsilon_{r}-1}{2}{\left(1+12\frac{h}{W}\right)}^{-\frac{1}{2}}$$

(5) $$\Delta L=0.412h\frac{\left(\varepsilon_{e}+0.3)(\frac{W}{h}+0.264\right)}{\left(\varepsilon_{e}-0.258)(\frac{W}{h}+0.8\right)}$$

**Figure 2:**
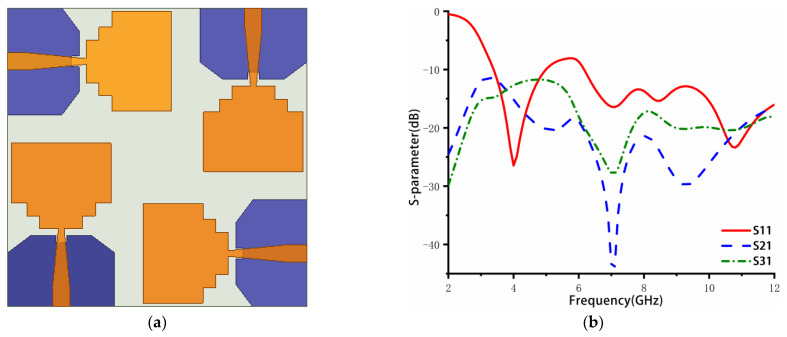
(**a**) Structure, (**b**) S parameters.

The four-element MIMO antenna is constructed based on the antenna element from step 4. As shown in Figure 2a, four elements are placed on the substrate orthogonally, and polarization diversity is used to improve the isolation between adjacent components. Its S-parameters are simulated and shown in Figure 2b. The return loss is only slightly greater than −10 dB from 5 to 6.1 GHz. Good isolation is obtained, with below −20 dB in the medium and high frequencies and below −12 dB in the low-frequency band. Therefore, further optimization and structures are needed to improve the bandwidth and isolation.

**Figure 3:**
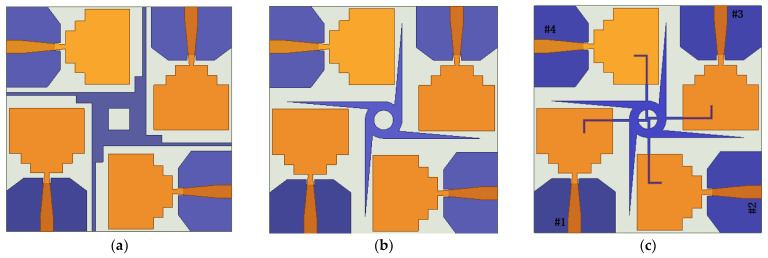
Evolution process of decoupling structure of MIMO antenna: (**a**) Three-step ladder structure, (**b**) windmill structure, (**c**) extended cruciform rotating parasitic branch structure.

**Figure 4:**
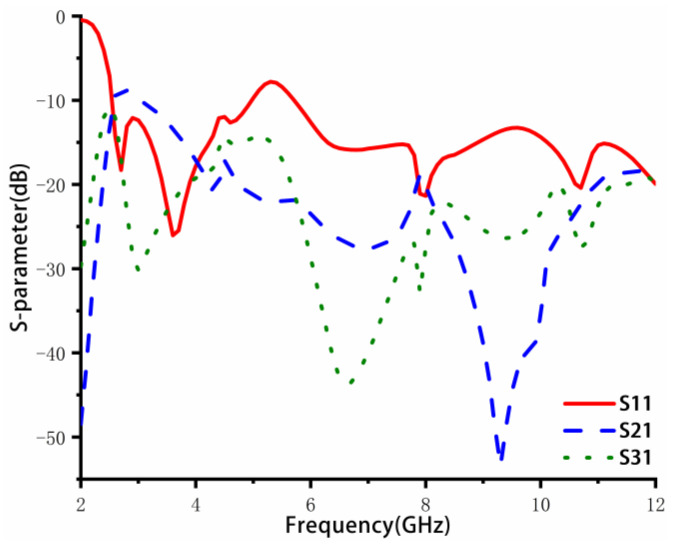
Simulation S parameters of three-step decoupling structure MIMO antenna.

There are two common coupling modes of MIMO antennas: Surface wave coupling and space wave coupling. In order to improve the decoupling between the MIMO antenna elements, as shown in Figure 3a, a four-directional three-stepped impedance converter is added to the ground. The simulated S-parameters are shown in Figure 4. Compared with the results in Figure 2b, the lower-frequency band of S11 is reduced to 2.9 GHz. At the same time, S21 and S31 at the middle and high frequencies have been further improved.

**Figure 5:**
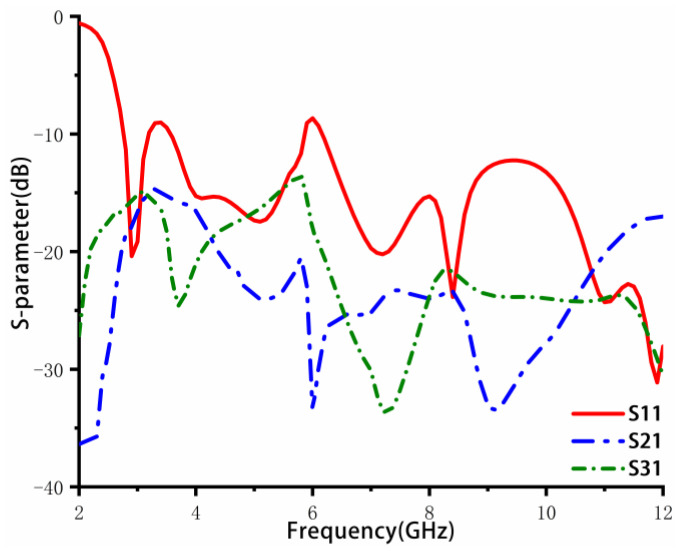
Windmill type decoupling structure MIMO antenna simulation S parameters.

In order to improve the return loss in the middle-frequency band at approximately 5–6 GHz, it is necessary to increase the order of the stepped impedance transformer to optimize the impedance matching. As the order increases to infinity, the converter slowly transforms into an asymptote-shaped structure, which looks similar to a windmill, as shown in Figure 3b. Figure 5 shows the simulated return loss and isolation of the design in Figure 3b. It can be seen that the antenna bandwidth is extended to 2.4–12 GHz, covering the whole UWB frequency band. Furthermore, S21 and S31 are less than −15 dB at the low-frequency band and less than −20 dB at the middle- and high-frequency bands. However, due to the coupling between the windmill-shaped structure and antenna elements, the S11 near 6 GHz is slightly higher than −10 dB. In addition, to achieve better isolation in the low-frequency band, the design in Figure 3c is finally proposed.

**Figure 6:**
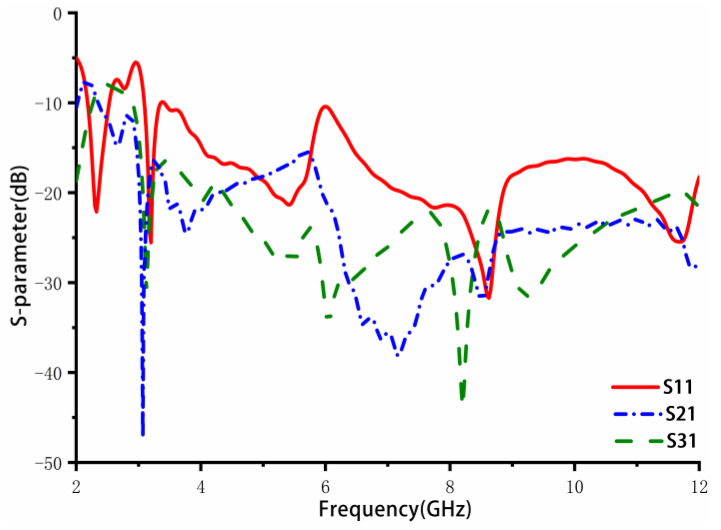
Simulation S parameters of the proposed four-element MIMO antenna.

Based on Figure 3b, a cross-shaped rotating branch is parasitically appended to the windmill-shaped structure. The S-parameters results are further improved, as shown in Figure 6. The surface wave coupling and space wave coupling currents are generated between adjacent elements and decoupling structures, offsetting each other after passing through the parasitic rotating branches. As a result, the coupling between adjacent antenna elements is reduced. The bandwidth of the antenna is from 3.09 to 12 GHz. The isolation is less than −16.4 dB in the low-frequency band and less than −20 dB in the middle- and high-frequency bands.

**Figure 7:**
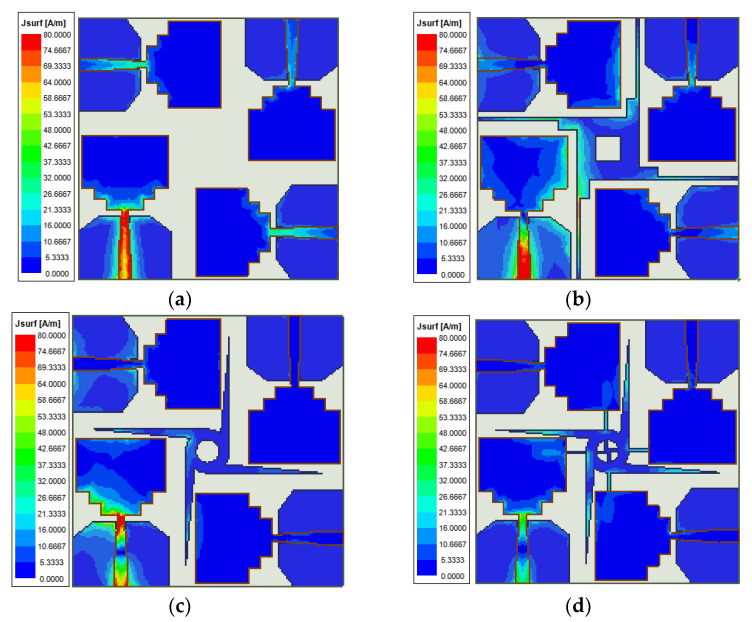
Simulation current distribution of MIMO antenna system; (**a**) polarization orthogonal decoupling structure, (**b**) three-step ladder decoupling structure, (**c**) windmill decoupling structure, and (**d**) cross-shaped rotary branch decoupling structure.

The surface current distribution of the antenna is shown at 3.5 GHz in Figure 7. When one of the elements is excited, the others are connected to 50 Ω match loads. It can be observed how the decoupling structures help the decoupling between the antenna elements. There are considerable surface currents on the other three radiation patches in Figure 7a. In Figure 7b,c, the currents become small gradually with the windmill shape added on the ground side. At last, in Figure 7d, there are several currents when the cross-shaped rotating branches are added.

**Figure 8:**
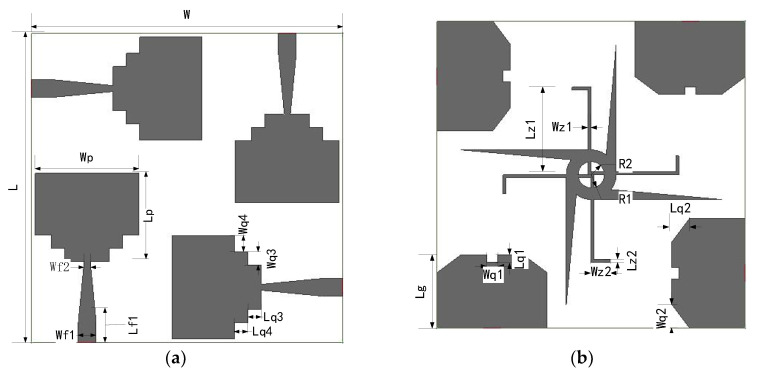
The schematic diagram of the proposed antenna: (**a**) Top, (**b**) bottom.

Figure 8 shows the geometry and dimensions of the proposed UWB-MIMO antenna. The overall size of the proposed antenna is 42 mm × 42 mm × 1 mm, printed on an FR4 substrate with a relative dielectric constant of 4.4. The antenna is simulated and optimized by ANSYS Electronics Desktop 2021. The geometry parameters are given in Table 1.

## 3. Results and Discussions

### 3.1. Fabrication and Measurement

**Figure 9:**
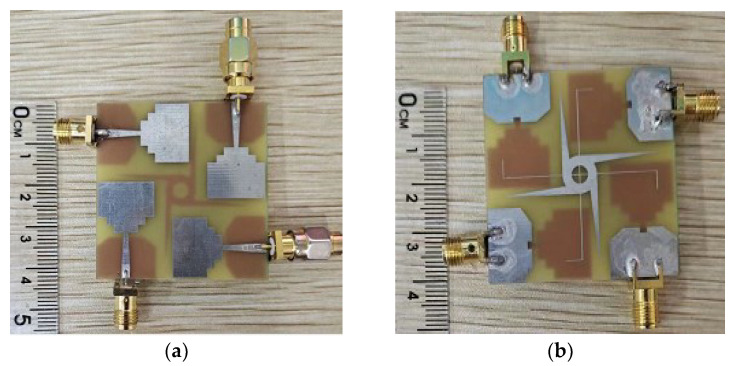
Fabricated four-element MIMO antenna prototype: (**a**) Top view, (**b**) bottom view.

**Figure 10:**
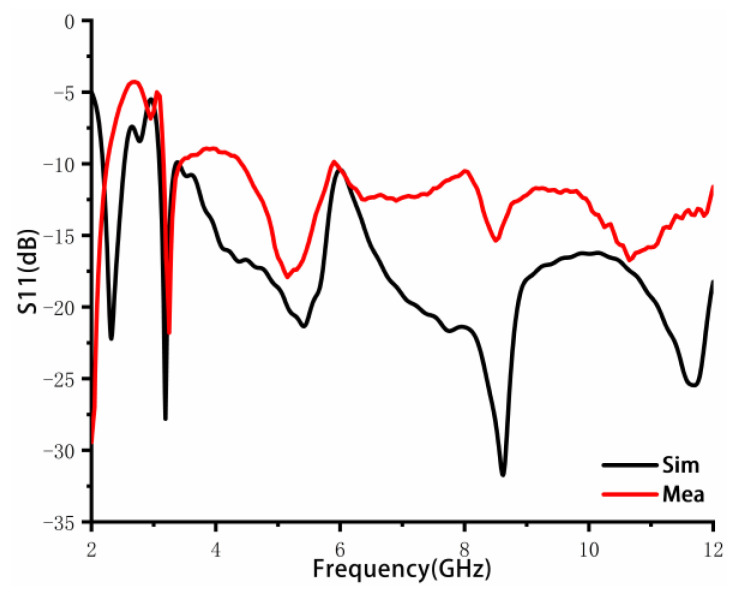
Simulated and measured return loss (S11).

To verify the feasibility and effectiveness of the proposed design antenna in Figure 3c, the antenna is fabricated. The photograph of the prototype is shown in Figure 9. The S-parameters of the antenna are measured using the ZVA 24 vector network analyzer of ROHDE&SCHWARZ Company. The simulated and measured return loss results are shown in Figure 10. The simulated frequency bandwidth agrees with the measured one, which is from 3.09 to 12 GHz. The small discrepancy is likely caused by the errors in the antenna fabrication, the welding errors of SMA joints, and the high loss of the FR4 dielectric substrate at high frequencies.

**Figure 11:**
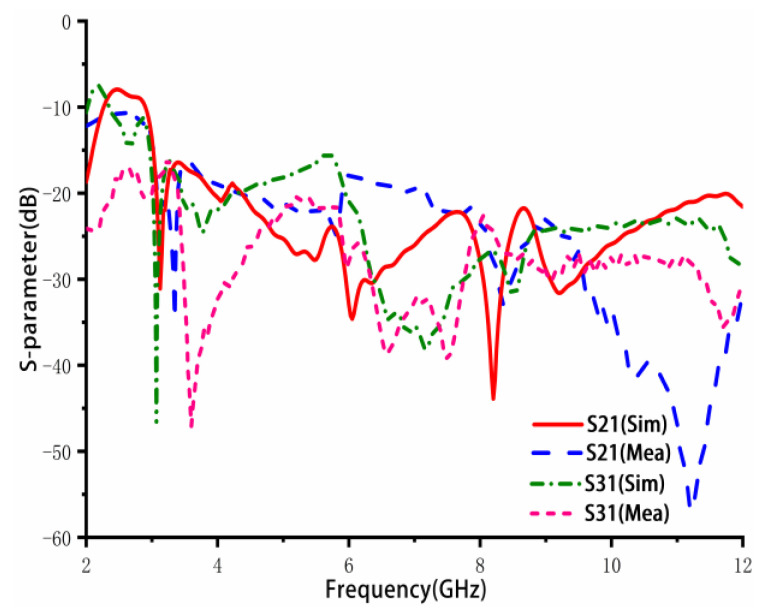
Simulated and measured isolation (S21, S31).

Figure 11 illustrates the simulated and measured results for the isolation between port 1 and port 2, and between port 1 and port 3. The results demonstrate that the isolation is less than −16.4 dB in the low-frequency band, less than −20 dB in the middle- and high-frequency bands, and even reaches −40 dB in some frequency bands, indicating a good isolation performance.

### 3.2. MIMO System Parameters

To evaluate the performance of the proposed antenna, several important parameters are investigated and analyzed. The envelope correlation coefficient (ECC) [31] is used to assess the degree of independence between antenna units and should be less than 0.5 for communication systems. Equation (6) is used to calculate the ECC, as shown below:

(6) $$\mathrm{ECC}=\frac{|S_{ii}^{*}S_{ij}+S_{ji}^{*}S_{jj}{|}^{2}}{\left|(1-|S_{ii}{|}^{2}-|S_{ji}{|}^{2})\cdot (1-|S_{jj}{|}^{2}-|S_{ij}{|}^{2})\right|}$$

Another important indicator for channel fading in MIMO antennas is diversity gain (DG) [32], which is calculated using Equation (7):

(7) $$\mathrm{DG}=10\sqrt{1-{\left(\mathrm{ECC}\right)}^{2}}$$

**Figure 12:**
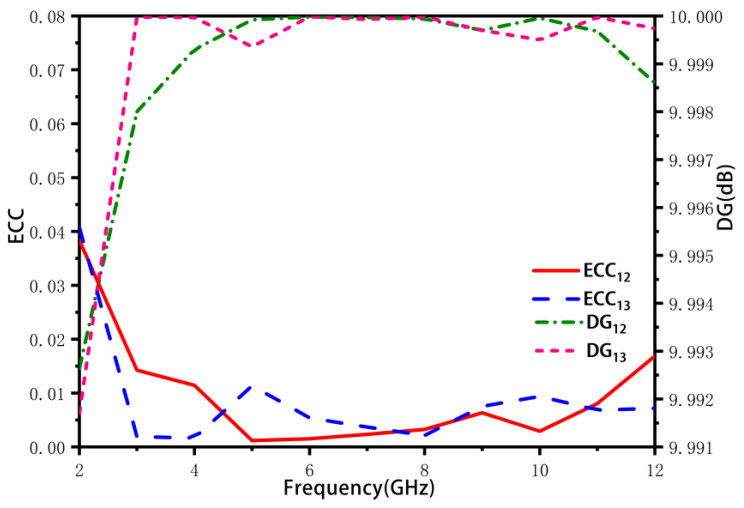
Line Port 1 and Port 2, Port 1 and Port 3 Analog ECC and DG.

Figure 12 displays the ECC and DG between port 1 and port 2, and between port 1 and port 3. The ECC is less than 0.02 and the DG is larger than 9.99 dB across the entire operating frequency band, indicating that the correlation between antenna elements is very small and meets the requirements of the MIMO system.

The total effective reflection coefficient (TARC) is defined as the ratio of the square root of the total reflected power to the square root of the total incident power, representing the mutual coupling between ports and the combination of random signals. Equation (8) is used to calculate TARC for a four-port MIMO antenna system [33]:

(8) $$\mathrm{TARC}=\frac{\sqrt{\begin{array}{l} |(S_{11}+S_{12}e^{j\theta }+S_{13}e^{j\theta^{\prime }}+S_{14}e^{j\theta^{\prime \prime }}){|}^{2}+|(S_{21}+S_{22}e^{j\theta }+S_{23}e^{j\theta^{\prime }}+S_{24}e^{j\theta^{\prime \prime }}){|}^{2}\\ +|(S_{31}+S_{32}e^{j\theta }+S_{33}e^{j\theta^{\prime }}+S_{34}e^{j\theta^{\prime \prime }}){|}^{2}+|(S_{41}+S_{42}e^{j\theta }+S_{43}e^{j\theta^{\prime }}+S_{44}e^{j\theta^{\prime \prime }}){|}^{2} \end{array}}}{2}$$

**Figure 13:**
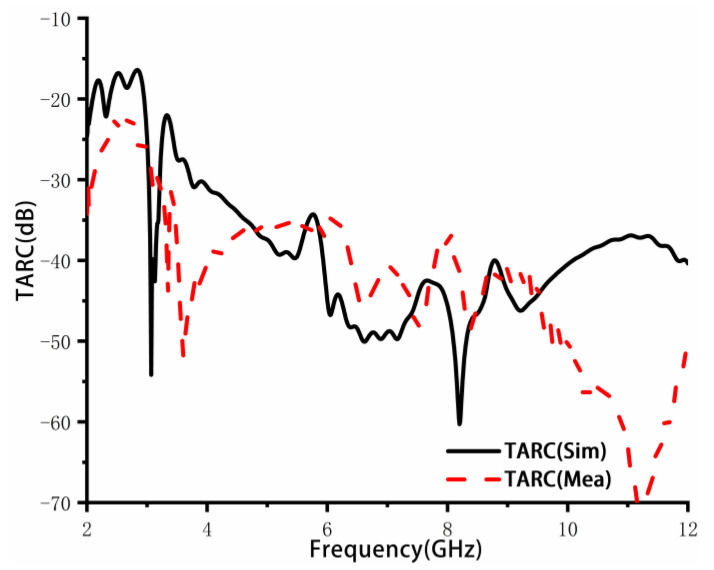
Simulated and measured TARC.

Figure 13 shows the TARC results, which were obtained by selecting nine combinations of random phases (θ, θ′, θ″) and calculating their average values. In a communication system, the TARC value of the antenna should be less than 0 dB. The measured average TARC in the operating frequency band is less than −20 dB, indicating that the proposed antenna is insensitive to phase changes and has a good bandwidth.

**Figure 14:**
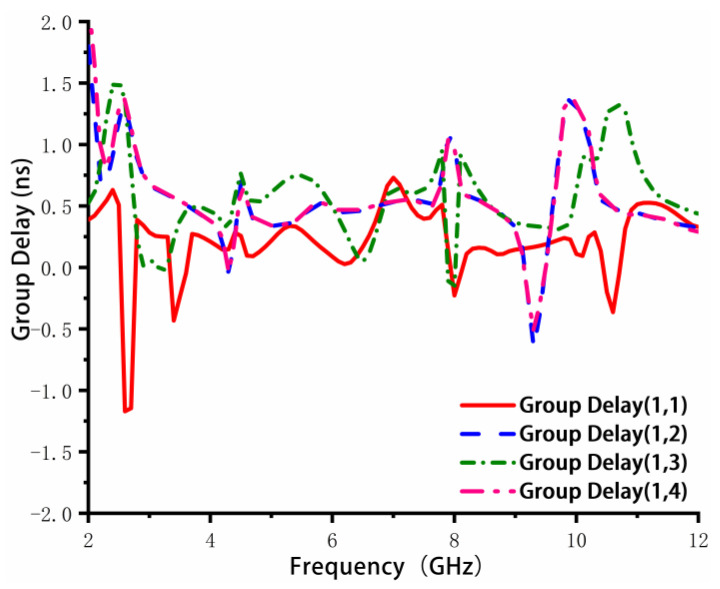
Group delay of the proposed MIMO antenna.

Group delay is an important time-domain characteristic of MIMO antennas [34]. It refers to the delay generated by the signal as a whole when the group signal passes through a linear system or network, which is the propagation time of the synthetic wave envelope. Therefore, it is also known as the envelope delay. Figure 14 illustrates the group delay variation of the proposed MIMO antenna in the operating band. Group delay (1,1) denotes the delay from port 1 to port 1, Group delay (1,2) indicates the delay from port 1 to port 2, and group delay (1,3) and (1,4) have similar definitions. Since the elements in the proposed MIMO antenna are identical and symmetrical, the group delay (1,2) and group delay (1,4) are almost similar. As shown in Figure 14, the variations in group delay are higher for the high operating spectrum. The total group delay value of the proposed MIMO antenna is less than 1.4 ns.

### 3.3. Far-Field Radiation Characteristics

**Figure 15:**
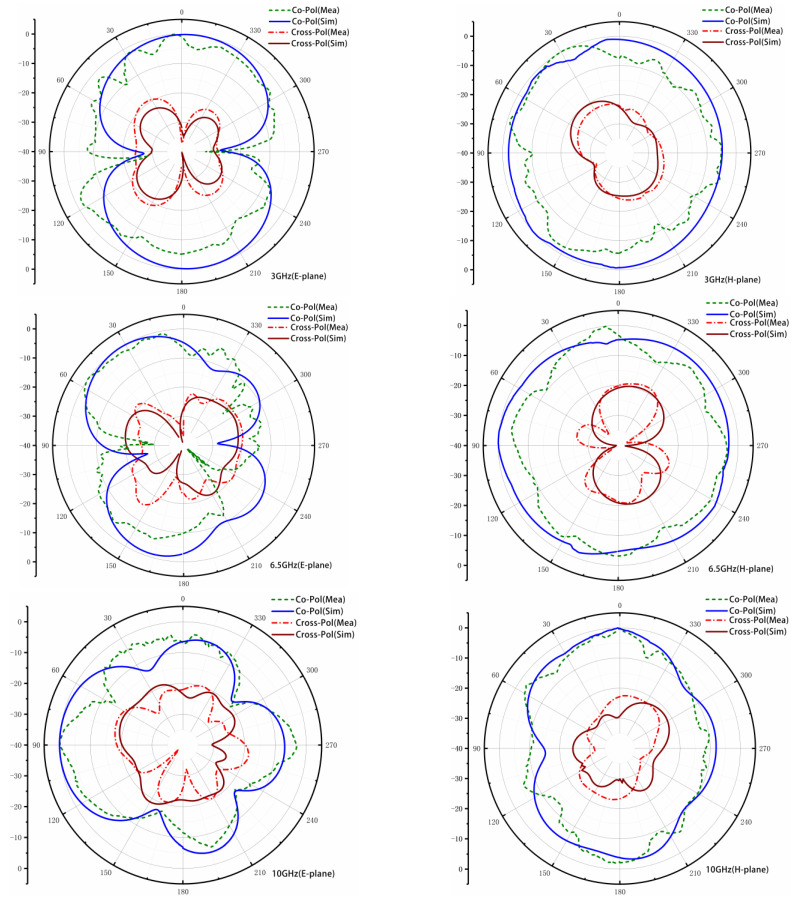
Simulated and measured radiation patterns of the proposed antenna in E-plane and H-plane.

The far-field results are obtained in the microwave anechoic chamber, with the excited port connected to the testing cable and the other ports terminated to 50 Ω matched loads. Figure 15 presents the simulated and measured co-polarization and cross-polarization radiation patterns of the proposed UWB-MIMO antenna in the E-plane and H-plane at 3, 6.5, and 10 GHz, respectively. As shown in Figure 15, the radiation mode of the antenna is relatively stable at low frequencies, whereas the radiation pattern of the antenna is distorted in the middle and high frequencies. It has been observed that the E-plane co-polarizations are directional, and the cross-polarizations are small, less than −20 dB in the main radiation directions. It has also been observed that the H-plane co-polarizations are quasi-omnidirectional and the cross-polarizations are small, less than 15 dB than co-polarizations. The measured results of the antenna radiation pattern are in good agreement with the simulated ones.

**Figure 16:**
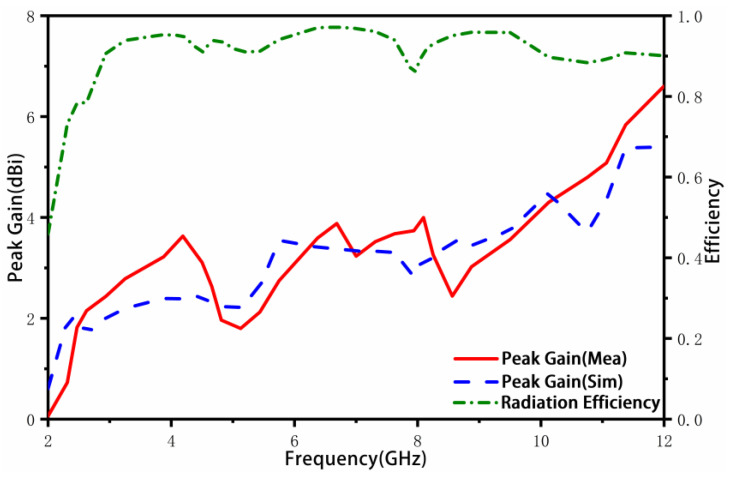
Simulation and measured peak gain and efficiency.

Figure 16 illustrates the simulated and measured results of peak gain and radiation efficiency of the proposed UWB-MIMO antenna. The peak gain ranges from 2 to 5.1 dBi within the operating frequency band, while the radiation efficiency is greater than 82%, indicating stable radiation characteristics.

Table 2 provides a summary and comparison of the antenna performance of the proposed design with other recent research studies. Compared with previous designs [9,35], the proposed antenna demonstrates a wider bandwidth. Additionally, it exhibits better isolation than [36,37] and a smaller size than [38,39]. Overall, considering its size, bandwidth, isolation, and cost, the proposed antenna offers several advantages [40].

The proposed antenna design incorporates several optimization techniques, as discussed in Section 2, including a stepped rectangular radiation patch to reduce antenna size, an asymptote-shaped microstrip-feeder to broaden bandwidth, and decoupling structures in the form of a windmill shape and a rotating extended cross shape to enhance antenna isolation. These optimization methods result in a compact antenna with wide bandwidth and excellent isolation characteristics. However, the main drawback of the proposed design is its relatively complicated geometry, as it adopts asymptote-shaped structures in several places.

## 4. Conclusions

In this paper, a compact four-element UWB-MIMO antenna with high isolation and ultrawide impedance bandwidth is proposed. Asymptote-shaped structures are introduced in the radiation patch, microstrip feeder, and decoupling structure to achieve a miniaturized size with a wide band and high isolation. A prototype of the proposed antenna is fabricated, and its S parameters, MIMO diversity characteristics, radiation pattern, peak gain, and other parameters are investigated through simulation and measurement. The results show good agreement between the simulated and measured values. The antenna operates over a frequency range of 3.09–12 GHz, has overall dimensions of 42 × 42 mm^2^ (0.43λ×0.43λ@ 3.09 GHz), and achieves isolation of less than −16.4 dB, making it compact and highly isolated. The antenna also exhibits good radiation and MIMO diversity characteristics, with an ECC less than 0.02, a DG larger than 9.991 dB, a TARC less than −20 dB, and group delay values less than 1.4 ns for the entire operating spectrum.

With the above characteristics, the proposed design has several advantages that make it well-suited for various applications, including:

- Wide bandwidth: The antenna has the ability to operate over a wide frequency range, enabling it to support high data rates and a large number of applications.
- Multipath mitigation: MIMO technology can effectively mitigate the effects of multipath fading, which is a common problem in wireless communication systems. This can lead to improved signal quality and higher data rates.
- Interference resistance: UWB technology is known for its ability to resist interference, making the proposed design well-suited for use in crowded environments where multiple wireless devices are in use.

Overall, the combination of UWB technology and MIMO technology in a single antenna provides several advantages that make the proposed antenna well-suited for applications such as WPANs, WSNs, and indoor positioning and tracking.

## Figures and Tables

**Table 1:** Optimized parameters of proposed UWB-MIMO antenna.

Parameters	Dimensions (mm)	Parameters	Dimensions (mm)	Parameters	Dimensions (mm)
W	42	Wf2	1	Wq4	2.2
L	42	Lf1	3.2	R1	3.4
H	1	Lq1	1	R2	1.8
Wp	14	Wq1	1.56	Lj	17.85
Lp	12	Lq2	2.4	Lz1	17
Lg	10	Wq2	3.2	Wz1	0.4
Wg	15	Lq3	1.8	Lz2	0.4
g	1	Wq3	4	Wz2	3.6
Wf1	2	Lq4	1.8		

**Table 2:** Comparison of several ultrawide-band MIMO antennas in recent studies.

Refs.	Substrate	Size $mm^{3}/\lambda_{0}{\;}^{1}$ $mm^{3}$	Operating Frequency (GHz)	Gain (dBi)	Isolation (dB)	ECC
[7]	Neltec	38×38×0.762 $\left(0.38\lambda_{0}\times 0.38\lambda_{0}\times 0.0076\lambda_{0}\right)$	3∼15	0.5∼5	>15	<0.5
[9]	FR4	60 × 60 × 1.6 $\left(0.6\lambda_{0}\times 0.6\lambda_{0}\times 0.016\lambda_{0}\right)$	3∼11	>3.4	>20	<0.02
[27]	FR4	58 × 58 × 0.8 $\left(0.73\lambda_{0}\times 0.88\lambda_{0}\times 0.008\lambda_{0}\right)$	3∼13.5	2.2∼4	>22	<0.008
[29]	Rogers RT/duriod5880	16 × 71.5 × 0.254 $\left(0.17\lambda_{0}\times 0.76\lambda_{0}\times 0.0027\lambda_{0}\right)$	3.2∼14	3∼5.6	>22	<0.006
[30]	Taconic RF-45	38.3 × 38.3 × 0.8 $\left(0.38\lambda_{0}\times 0.38\lambda_{0}\times 0.008\lambda_{0}\right)$	3∼13.2	0.5∼6.3	>17	<0.03
[35]	FR4	50 × 50 × 1.6 $\left(0.52\lambda_{0}\times 0.52\lambda_{0}\times 0.0165\lambda_{0}\right)$	3.1∼10.6	2∼6	>17	<0.02
[36]	FR4	56.1 × 67.9 × 2.3 $\left(0.73\lambda_{0}\times 0.88\lambda_{0}\times 0.03\lambda_{0}\right)$	3.89∼17.09	3.4∼6.8	>15	<0.02
[37]	FR4	42 × 42 × 1.6 $\left(0.42\lambda_{0}\times 0.42\lambda_{0}\times 0.016\lambda_{0}\right)$	3∼11	3∼4.5	>15	<0.05
[38]	FR4	65 × 65 × 1.6 $\left(0.67\lambda_{0}\times 0.67\lambda_{0}\times 0.0165\lambda_{0}\right)$	3.1∼10.6	N/A	>15	<0.025
[39]	FR4	75.19 × 75.19 × 1.6 $\left(0.78\lambda_{0}\times 0.78\lambda_{0}\times 0.0165\lambda_{0}\right)$	3.1∼17.3	1∼5	>15	<0.1
Prop.	FR4	42 × 42 × 1 $\left(0.43\lambda_{0}\times 0.43\lambda_{0}\times 0.0103\lambda_{0}\right)$	3.09∼12	2∼5.1	>16.4	<0.02

^1^*λ*_0_ represents the wavelength in the air at the lowest frequency.

## Data Availability

Not applicable.

## References

[1] Powell J., Chandrakasan A. Differential and single ended elliptical antennas for 3.1–10.6 GHz ultrawideband communication. Proceedings of the Antennas and Propagation Society International Symposium.

[2] El-Hameed A.S.A., Wahab M.G., Elshafey N.A., Elpeltagy M.S. (2021). Quad-port UWB MIMO antenna based on LPF with vast rejection band. AEU-Int. J. Electron. Commun..

[3] Manoharan H., Selvarajan S., Yafoz A., Alterazi H.A., Chen C. (2022). Deep Conviction Systems for Biomedical Applications Using Intuiting Procedures with Cross Point Approach. Front. Public Health.

[4] Iqbal A., Smida A., Alazemi A.J., Waly M.I., Mallat N.K., Kim S. (2020). Wideband Circularly Polarized MIMO Antenna for High Data Wearable Biotelemetric Devices. IEEE Access.

[5] Ibrahim A.A., Abdalla M.A., Abdel-Rahman A.B., Hamed H.F. (2014). Compact MIMO Antenna with Optimized Mutual Coupling Reduction Using DGS. Int. J. Microw. Wirel. Technol..

[6] Toktas A., Akdagli A. (2015). Compact multiple-input multiple-output antenna with low correlation for ultra-wide-band applications. IET Microw. Antennas Propag..

[7] Sipal D., Abegaonkar M.P., Koul S.K. (2017). Easily Extendable Compact Planar UWB MIMO Antenna Array. IEEE Antennas Wirel. Propag. Lett..

[8] Khan M.S., Capobianco A.D., Asif S., Iftikhar A., Braaten B.D. A 4 element compact Ultra-Wideband MIMO antenna array. Proceedings of the IEEE International Symposium on Antennas & Propagation & USNC/URSI National Radio Science Meeting.

[9] Ahmad S., Khan S., Manzoor B., Soruri M., Alibakhshikenari M., Dalarsson M., Falcone F. (2022). A Compact CPW-Fed Ultra-Wideband Multi-Input-Multi-Output (MIMO) Antenna for Wireless Communication Networks. IEEE Access.

[10] Tang Z., Wu X., Zhan J., Hu S., Xi Z., Liu Y. (2019). Compact UWB-MIMO Antenna with High Isolation and Triple Band-Notched Characteristics. IEEE Access.

[11] Chen Z., Zhou W., Hong J. (2021). A Miniaturized MIMO Antenna with Triple Band-Notched Characteristics for UWB Applications. IEEE Access.

[12] Abdelhamid C., Daghari M., Sakli H., Hamrouni C. High Isolation with Metamaterial Improvement in A Compact UWB MIMO Multi-Antennas. Proceedings of the 2019 16th International Multi-Conference on Systems, Signals & Devices (SSD).

[13] Khan A., Bashir S., Ghafoor S., Qureshi K.K. (2021). Mutual Coupling Reduction Using Ground Stub and EBG in a Compact Wideband MIMO-Antenna. IEEE Access.

[14] Zhang S., Pedersen G.F. (2016). Mutual Coupling Reduction for UWB MIMO Antennas with a Wideband Neutralization Line. IEEE Antennas Wirel. Propag. Lett..

[15] Wang S.-L., Hong J.-S., Wang C., He J.-F. A nonplanar quad-element UWB-MIMO antenna with graphite sheet to increase the isolation. Proceedings of the 2018 IEEE MTT-S International Wireless Symposium (IWS).

[16] Khan M.S., Capobianco A.-D., Asif S.M., Anagnostou D.E., Shubair R.M., Braaten B.D. (2017). A Compact CSRR-Enabled UWB Diversity Antenna. IEEE Antennas Wirel. Propag. Lett..

[17] Luo C.-M., Hong J.-S., Zhong L.-L. (2015). Isolation enhancement of a very compact UWB-MIMO slot antenna with two defected ground structures. IEEE Antennas Wirel. Propag. Lett..

[18] Anitha R., Sarin V.P., Mohanan P., Vasudevan K. (2014). Enhanced isolation with defected ground structure in MIMO antenna. Electron. Lett..

[19] Ren J., Hu W., Yin Y., Fan R. (2014). Compact Printed MIMO Antenna for UWB Applications. IEEE Antennas Wirel. Propag. Lett..

[20] Niu Z., Zhang H., Chen Q., Zhong T. (2019). Isolation Enhancement for 1 × 3 Closely Spaced E-Plane Patch Antenna Array Using Defect Ground Structure and Metal-Vias. IEEE Access.

[21] Ul HM A., Slawomir K. (2018). Ground Plane Alterations for Design of High-Isolation Compact Wideband MIMO Antenna. IEEE Access.

[22] Iqbal A., Saraereh O.A., Ahmad A.W., Bashir S. (2018). Mutual Coupling Reduction Using F-Shaped Stubs in UWB-MIMO Antenna. IEEE Access.

[23] Kulkarni J., Alharbi A.G., Elfergani I., Anguera J., Zebiri C., Rodriguez J. (2022). Dual Polarized, Multiband Four-Port Decagon Shaped Flexible MIMO Antenna for Next Generation Wireless Applications. IEEE Access.

[24] Girjashankar P.R., Upadhyaya T., Desai A. (2022). Multiband hybrid MIMO DRA for Sub-6 GHz 5G and WiFi-6 applications. Int. J. RF Microw. Comput.-Aided Eng..

[25] Satam V., Nema S. Dual polarized four element diversity antenna for UWB applications. Proceedings of the 2017 IEEE International Conference on Antenna Innovations & Modern Technologies for Ground, Aircraft and Satellite Applications (iAIM).

[26] Lin M., Li Z. A compact 4 × 4 dual band-notched UWB MIMO antenna with high isolation. Proceedings of the 2015 IEEE 6th International Symposium on Microwave, Antenna, Propagation, and EMC Technologies (MAPE).

[27] Raheja D.K., Kanaujia B.K., Kumar S. (2019). Compact four-port MIMO antenna on slotted-edge substrate with dual-band rejection characteristics. Int. J. RF Microw. Comput.-Aided Eng..

[28] Mishra B., Siddiqui R.A., Tripathy M.R. A Compact Four Elements MIMO antenna for UWB Application. Proceedings of the 2018 IEEE Indian Conference on Antennas and Propogation (InCAP).

[29] Rao P.K., Mishra R. (2020). Elliptical Shape Flexible MIMO Antenna with High Isolation for Breast Cancer Detection Application. IETE J. Res..

[30] Gómez-Villanueva R., Jardón-Aguilar H. (2019). Compact UWB Uniplanar Four-Port MIMO Antenna Array with Rejecting Band. IEEE Antennas Wirel. Propag. Lett..

[31] Blanch S., Romeu J., Corbella I. (2003). Exact presentation of antenna system diversity performance from input parameter description. Electron. Lett..

[32] Sharma M., Choudhary N., Kumar N., Panda S., Kaushal R. A Slotted Hexagonal 4 × 4 MIMO Antenna with Tapered feed Designed for High Speed IoT Wireless Applications. Proceedings of the 2021 6th International Conference on Signal Processing, Computing and Control (ISPCC).

[33] Chae S.H., Kawk W.I., Park S., Lee K. Analysis of mutual coupling in MIMO antenna array by TARC calculation. Proceedings of the Asia–Pacific Microwave Conference.

[34] Rekha V.S.D., Pardhasaradhi P., Madhav B.T.P., Devi Y.U. (2020). Dual band notched orthogonal 4-element MIMO antenna with isolation for UWB applications. IEEE Access.

[35] Keerthana G., Naidu P.V., Priyanka K., Sumanji L., Saiharanadh A., Maheshbabu D., Kumar A., Priyanka V. High Isolation Compact Four Port MIMO Antenna with Slotted Ground for UWB Applications. Proceedings of the 2021 Photonics & Electromagnetics Research Symposium (PIERS).

[36] Desai A., Kulkarni J., Kamruzzaman M.M., Hubálovský Š., Hsu H.-T., Ibrahim A.A. (2022). Interconnected CPW Fed Flexible 4-Port MIMO Antenna for UWB, X, and Ku Band Applications. IEEE Access.

[37] Mathur R., Dwari S. A compact 4-port UWB-MIMO/diversity antenna for WPAN application. Proceedings of the 2018 3rd International Conference on Microwave and Photonics (ICMAP).

[38] Lamri I.E., Mansoul A., Nakmouche M.F., Belattar M. Design of Novel UWB 4-element MIMO Microstrip Patch Antenna for Sub-6 GHz 5G Applications. Proceedings of the 2021 International Conference on Radar, Antenna, Microwave, Electronics, and Telecommunications (ICRAMET).

[39] Kayabasi A., Toktas A., Yigit E., Sabanci K. (2018). Triangular quad-port multi-polarized UWB MIMO antenna with enhanced isolation using neutralization ring. AEU-Int. J. Electron. Commun..

[40] Bing Y., Minzhe C., Lingyum L. (2018). Design of a four-element WLAN/LTE/UWB MIMO antenna using half-slot structure. AEU-Int. J. Electron. Commun..

